# Supplementary material for: Dynamic Hormone Gradients Regulate Wound-Induced de novo Organ Formation in Tomato Hypocotyl Explants
Source: Int J Mol Sci. 2021 Oct 31;22(21):11843. doi: 10.3390/ijms222111843 (PMC8584571; doi:10.3390/ijms222111843)
Supplement: Supplementary file 1 [file ijms-22-11843-s001.zip › 00_Larriba_IJMS2_supplemental_REVIEWED.pdf]

Figure S1

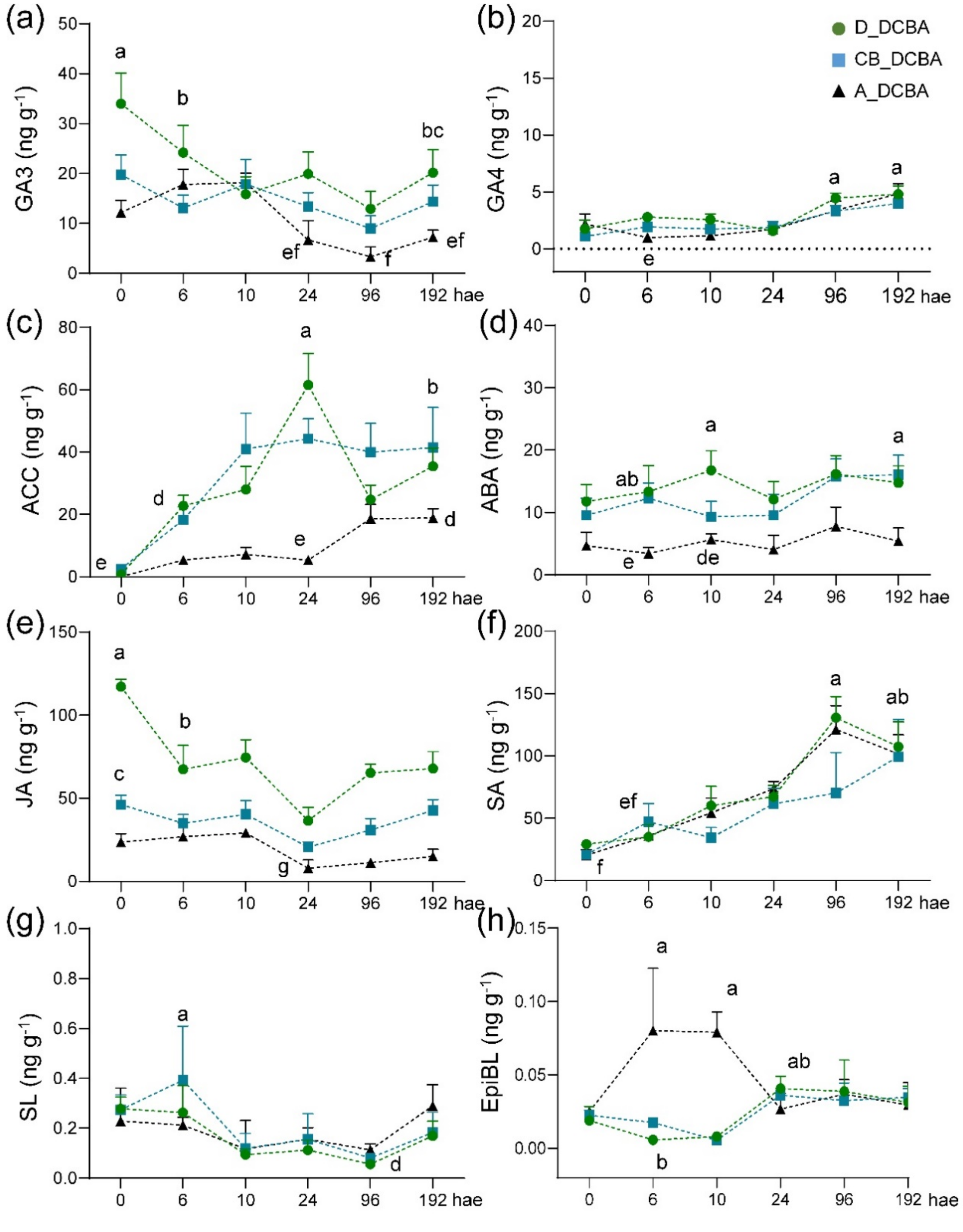

Figure S1. Endogenous hormone levels during de novo organ formation (cont.). (**a**) GA3, (**b**) GA4, (**c**) the ethylene precursor 1-aminocyclopropane-1-carboxylic acid (ACC), (**d**) abscisic acid (ABA), (**e**) jasmonic acid (JA), (**f**) salicylic acid (SA), (**g**) solanacol (SL), and (**h**) epibrassinolide (EpiBL). Different letters indicate significant differences (p-value<0.001) between samples.

Figure S2

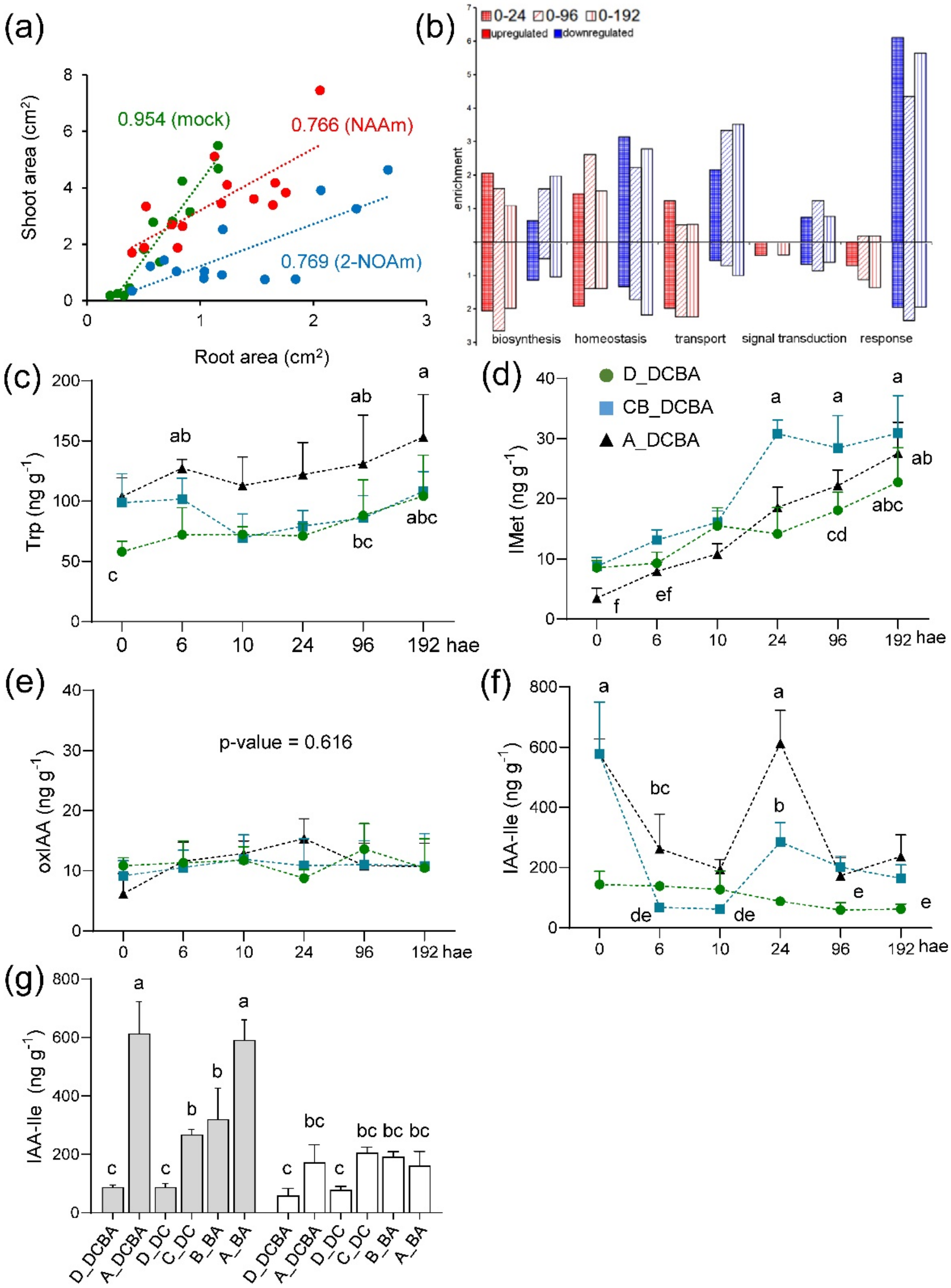

Figure S2. Endogenous levels of auxin metabolites during de novo organ formation. (**a**) Relationship between root area and shoot area at 21 dae. Numbers indicate Person's correlation coefficient in the studied samples. (**b**) Enrichment of DEGs in different auxin subcategories (see **Materials and Methods**). (**c-f**) Accumulation of auxin metabolites during de novo organ formation: (**c**) Tryptophan (Trp), (**d**) indole-3-methanol (IMet), (**e**) oxyndole-3-acetic acid (oxIAA), and (**f**) indole-3-acetyl-L-isoleucine (IAA-Ile). (**g**) Endogenous levels of IAA-Ile at 24 and 96 hae in apical (D) and basal (A) regions of whole (DCBA) or half-sectioned (DC and BA) hypocotyl explants. Different letters in c-g indicate significant differences (p-value<0.001, otherwise indicated) between samples.

Figure S3

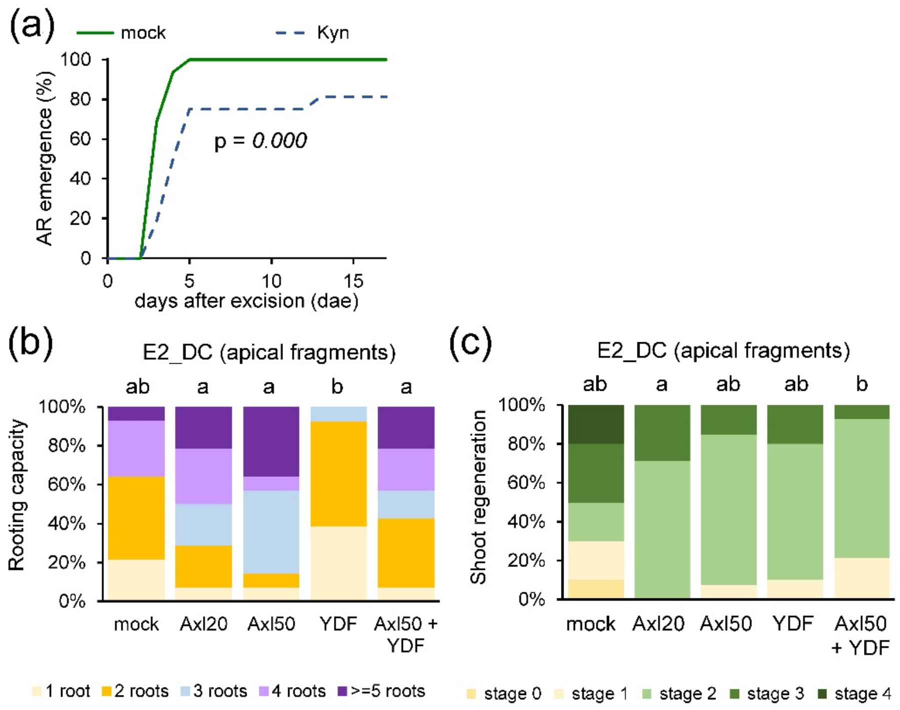

Figure S3. Auxin regulation during de novo organ formation (cont.). (**a**) AR emergence of whole hypocotyl explants in response to the indicated treatment; Kyn: 25 µM L-kynurenine added to the growing medium. (**b**) Rooting capacity of apical hypocotyl explants at 17 dae. (**c**) Shoot regeneration stages of 'Micro-Tom' explants at 17 dae. Explants in b, c were continuously incubated with 20 µM auxinole (Axl20), 50 µM auxinole (Auxl50), 50 µM yucasin DF (YDF), or 50 µM auxinole and 50 µM yucasin DF. Different letters in b, c indicate significant differences (p-value < 0.001) between treatments.

Figure S4

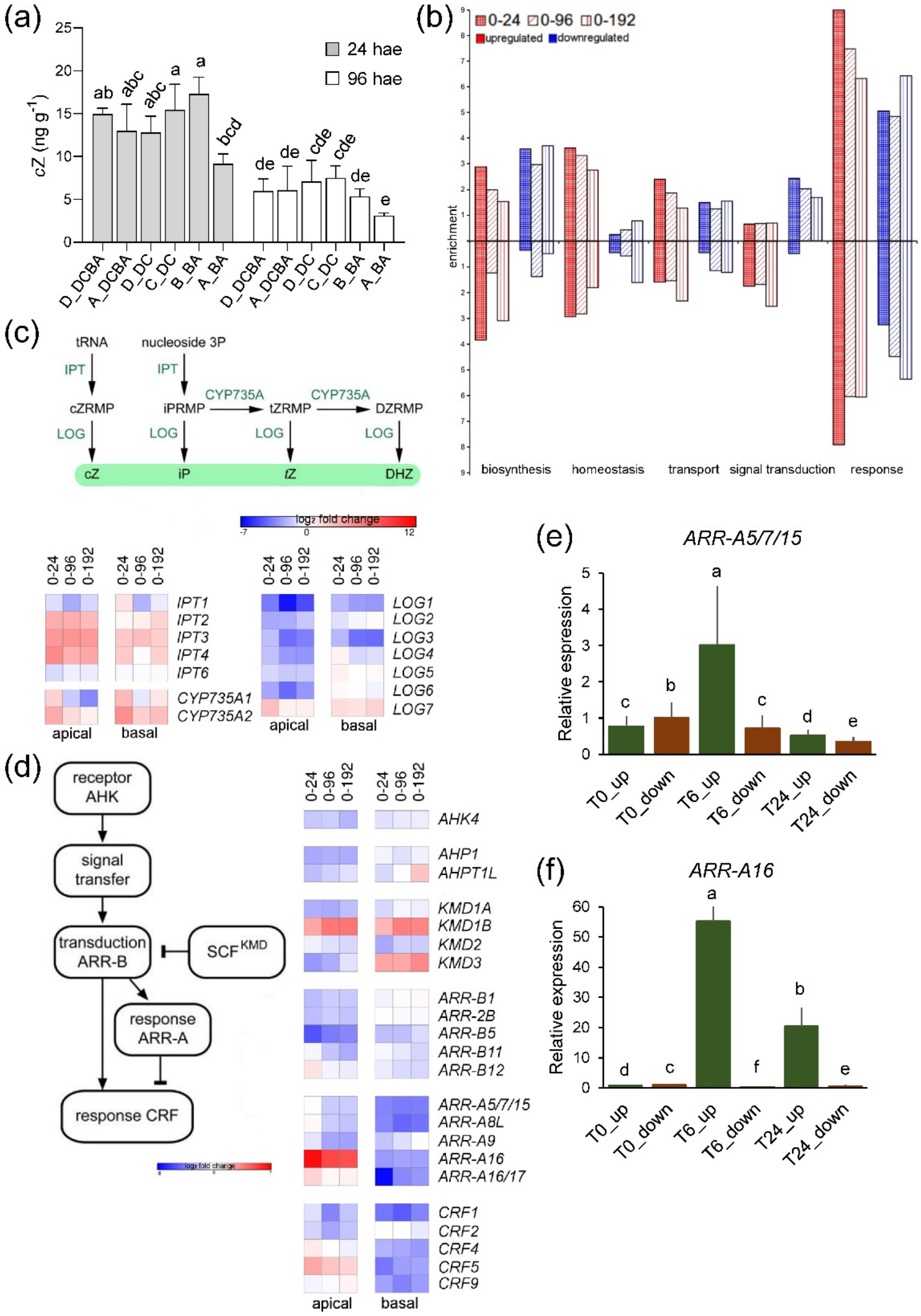

Figure S4. Regulation of CK pathway genes during de novo organ formation. (**a**) Endogenous levels of *cZ* at 24 and 96 hae in apical (D) and basal (A) regions of whole (DCBA) or half-sectioned (DC and BA) hypocotyl explants. (**b**) Enrichment of DEGs in different CK subcategories. Expression values of DEGs were in $\log_2$ fold change relative to 0 hae according to the scale bars in figure (FDR < 0.01). (**c**) Schematic diagram of CK biosynthesis and DEGs found in this pathway. (**d**) Schematic diagram of CK transduction pathway and DEGs found in this pathway. (**e-f**) Relative expression of *ARR-A5/7/15* (e) and *ARR-A16* (f) at 0, 6 and 24 hae in apical (green bars) and basal (brown bars) regions of the hypocotyl. Different letters in e, f indicate significant differences (p-value<0.001) between samples.

Figure S5

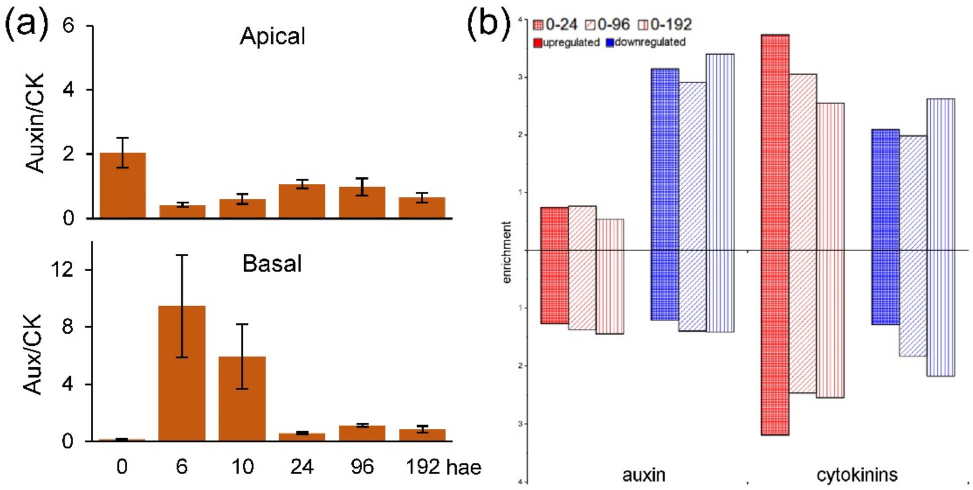

Figure S5. Auxin-to-CK ratio regulates de novo organ formation (cont.). (**a**) IAA-to-CK ratio in the apical (D) and the basal (A) region of whole (DCBA) hypocotyl explants during the studied time course. (**b**) Enrichment of DEGs in different auxin and CK subcategories (see **Materials and Methods**).

Table S1. Raw data for hormonal measurements (ng/mL). (**a**) Results from whole hypocotyl explants (DCBA fragments). (**b**) Results from half-sectioned hypocotyl explants (DC and BA fragments).

Table S2. Annotation and classification of genes associated to auxin biosynthesis, homeostasis, transport, and response in tomato. SolycID and ITAG4.0 annotation were retrieved from SolGenomics (https://solgenomics.net/). Putative *Arabidopsis thaliana* orthologs were identified from the Ensembl Plants database using BioMart. Legend: %id. Target A. thaliana, percentage of identity of target *A. thaliana* gene identical to tomato gene; %id. tomato gene, percentage of identity of target tomato gene identical to *A. thaliana* gene; confidence, orthology confidence score from the Emsembl Plants database. KEGG identifiers and annotations were retrieved using an RBBH strategy (see **Materials and Methods**).

Table S3. Annotation and classification of genes associated to CK biosynthesis, homeostasis, transport, and response in tomato. See legend from **Table S2**.

Table S4. Primers for qRT-PCR validation of RNA-seq data. Gene name and gene identifier in ITAG4.0 annotation (Solyc ID) are included. Nucleotide sequence of the forward and reverse primers and amplicon size (cDNA) are indicated.
